# Mortality From Respiratory Syncytial Virus in Children Under 2 Years of Age: A Prospective Community Cohort Study in Rural Maharashtra, India

**DOI:** 10.1093/cid/ciab481

**Published:** 2021-09-02

**Authors:** Eric A F Simões, Vibhawari Dani, Varsha Potdar, Rowena Crow, Shilpa Satav, Mandeep S Chadha, Danielle Hessong, Phyllis Carosone-Link, Sameer Palaskar, Ashish Satav

**Affiliations:** 1 Department of Paediatric Infectious Diseases, University of Colorado School of Medicine and Children’s Hospital Colorado Aurora, Colorado, USA; 2 Centre for Global Health, Department of Epidemiology,Colorado School of Public Health, Aurora, Colorado, USA; 3 MAHAN Trust Mahatma Gandhi Tribal Hospital, Karmgram, Utavali, Tahsil, Dharni, India; 4 National Institute of Virology, Indian Counsel of Medical Research, Pune, India

**Keywords:** acute lower respiratory infection (ALRI), infant mortality, epidemiology

## Abstract

**Background:**

Although respiratory syncytial virus (RSV) is the most important viral cause of lower respiratory tract infection deaths in infants, there are few data on infant community deaths caused by RSV.

**Methods:**

This was an active surveillance of children younger than 2 years of age in 93 villages, 5 primary health centers, and 3 hospitals serving these villages. Village health workers and counselors at the health facilities monitored all lower respiratory tract infections (LRTIs) in consented subjects. Children with severe, or very severe LRTIs and all who died, had nasopharyngeal swabs collected for detection of RSV by molecular methods.

**Results:**

In the 12 134 subjects, there were 2064 episodes of severe LRTIs and 1732 of very severe LRTIs, of which 271 and 195, respectively, had RSV. Fifteen of 16 (94%) children with RSV died of LRTIs, 14 in the community and 1 in the hospital. The case fatality ratios for severe RSV LRTIs in the first 6 months of life were 3/52 (7.1%) and 1/36 (2.8%) in the community and hospital, respectively. Of those with very severe LRTIs in the community, 17.6% died. There were no very severe RSV LRTI hospital deaths. The adjusted RSV LRTI mortality rates ranged from 1.0 to 3.0/1000 child-years (CY) overall, and 2.0 to 6.1/1000 CY, accounting for 20% of the LRTI deaths and 10% of the postneonatal infant mortality.

**Conclusions:**

Community deaths from RSV account for the majority of RSV LRTI deaths, and efforts at prevention should be preferentially directed at populations where access to care is limited.

## Teaser

Respiratory syncytial virus is the most important viral cause of lower respiratory tract infection deaths in infants, and efforts at prevention should be preferentially directed at populations where access to care is limited.

Respiratory syncytial virus (RSV) is the single most important cause of hospitalization and the most important viral cause of death from acute respiratory tract infections in young children globally. Most of these deaths occur in the community [1]. Recognizing that maternal immunization and other preventive modalities for RSV were on the horizon, the Bill and Melinda Gates Foundation funded a series of community-based studies in Argentina, India, Pakistan, and Zambia [2] as well as the use of minimally invasive tissue sampling in a large study in Africa and Asia, and the Child Health and Mortality Prevention Surveillance (CHAMPS) network [3]. These latter studies initially focused on hospital mortality, whereas the community-based studies focused primarily on the community.

India is the single largest country contributing to the large global burden of RSV disease in infants and young children as well as to mortality from the virus, mostly in rural areas [4]. Previous attempts at estimating this burden have relied primarily on hospital-based case fatality ratios (CFRs). Although there are numerous studies of in-hospital mortality from RSV, there are scant recent prospective studies to estimate the community and hospital burden of RSV disease, including mortality from it [1, 5]. To gain insight into both the hospital and community burden of RSV in children, we conducted a prospective community cohort study in rural Maharashtra, India, with the aim to gather morbidity and mortality data attributable to RSV.

## METHODS

### Study Design

This was a prospective active community and hospital surveillance study conducted in 93 tribal villages of Melghat, Central India, between 1 September 2016 and 31 March 2020. The methods described in detail in the accompanying article describing the morbidity from RSV are summarized in the following sections [6].

### Study Area

Melghat is a predominantly rural, mostly tribal, community located in the forested hilly areas in central Maharashtra. Two government district and subdistrict hospitals, a charitable trust hospital (MAHAN), and 5 primary health centers provide the majority of facility-based care for the 93 villages.

### Obtaining Clearances

Before any research procedures, clearances were obtained from the Government of India (Indian Council of Medical Research), the Government of Maharashtra, the National Institute of Virology (located in Pune, India), Colorado Multiple Institutional Review Board of the University of Colorado, and MAHAN Institutional Review Board Clearance. We approached 100 villages for inclusion in the study, of which 93 villages consented to the study. Village health workers (VHWs) living in the villages, usually 1 per village, were the main field workers for the study.

### Study Population

In September 2016, trained VHWs and 1 supervisor per 10 villages conducted a census of all households in the villages. All children younger than age 2 years were recruited for prospective observation for severe lower respiratory tract infections (LRTIs) after obtaining informed consent from the parent/caretaker. Subsequently using pregnancy monitoring, all parents of live-born neonates were approached for consent to follow-up their infant for the next 2 years. Subjects were followed until they reached age 2 years, migrated permanently out of the 93 villages, withdrew consent, or expired.

### Health Worker Training

All VHWs and their supervisors and health center-/hospital-based counselors were trained using a modified World Health Organization (WHO) methodology, in the recognition of WHO-defined pneumonia [7], and its recording on paper case report forms as well as how to collect nasopharyngeal (NP) swabs in infants and children. WHO classifications included nonsevere, severe, and very severe categories of pneumonia, (Supplementary Table 1). VHWs were trained in survey taking, as well as in filling out weekly reports on the health of subjects, pregnancy monitoring, and monitoring for subject deaths. Counselors were trained in filling out case report forms for hospital admissions, and supervisors were trained in grief counselling and conducting verbal autopsies using a standardized semistructured form.

### Subject Monitoring in the Community

VHWs developed a keen rapport with the families they were following, with weekly home visits, specifically following the respiratory health of the subjects. They monitored children for the development of WHO-defined pneumonia (LRTI). Any child with an LRTI, if severe or very severe, had an NP swab collected for pathogen detection, and then they were referred to either of the 2 hospitals. Within 24 hours of the assessment, supervisors visited the subject and conducted a respiratory assessment as well. All subjects with severe or very severe pneumonia who were hospitalized were followed up at home 1 and 2 weeks later.

VHWs were also informed of community deaths and, after grief counselling was offered to the family, either by a supervisor or local trained traditional healers or nurses, with informed consent, NP swabs were obtained from the deceased child whenever feasible.

### Subject Monitoring in the Health Centers and Hospitals

Counsellors working in the 7 government facilities conducted the daily census of all new admissions and, after obtaining informed consent, administered a short questionnaire and collected an NP sample from most children with LRTI or sepsis.

### NP Swab Collection and Sample Processing

NP swabs were collected using flocked swabs and transported in PrimStore MTM, (Longhorn Vaccines & Diagnostics, Bethesda, MD) to MAHAN hospital where they were stored at 4–80°C. Batches were transported to ICMR, National Institute of Virology Pune, for pathogen testing. The samples were tested for RSV and other respiratory viruses by real-time polymerase chain reaction using standardized protocols [8, 9].

### Verbal Autopsy

A supervisor and a VHW from the community conducted a verbal autopsy (VA) within 2 weeks of the death. The VA used standardized Indian government protocols modified for use in tribal areas of Maharashtra incorporating local terminology [10, 11]. The narratives were read by 2 independent trained physicians and, where there was discordance, a third senior pediatrician adjudicated the difference.

### LRTI: Cause of Death

A child with an LRTI classified either by a VHW or supervisor in the village or physician in a hospital who died within 15 days of the diagnosis was considered an LRTI death. In the absence of an examination, a verbal autopsy classified as pneumonia codified the death as an LRTI death.

### Data Entry

Data entry operators entered data into an access database and the data manager checked data quality. Incomplete data forms were sent back to the field for correction. The Indian principal investigator (A.S.) sent data to University of Colorado Denver for analysis and monthly reviews.

### Statistical Methods

The RSV season was determined separately for each season, commencing with the week of the first seasonal case of RSV and ending with the week of the last seasonal case of RSV. NP swab collection was attempted for all child deaths. The RSV rate among those with LRTI deaths grouped by age with NP swabs collected was applied to the deaths by age group, where swabs were not collected, to derive an adjusted death rate because of RSV. Rates of RSV were computed per 1000 child-years (CY) of observation. Ninety-five percent confidence intervals of the rates were computed using a Poisson approximation to the binomial distribution [12]. Differences in demographics were tested for significance using a Cochran’s Mantel-Haenszel χ ^2^ test for categorical variables, and *t* tests for continuous variables. Where the *t* test assumptions were not met, a nonparametric Wilcoxon rank-sum test was used. Wealth scores were computed as described elsewhere [6].

## RESULTS

**Figure 1.**
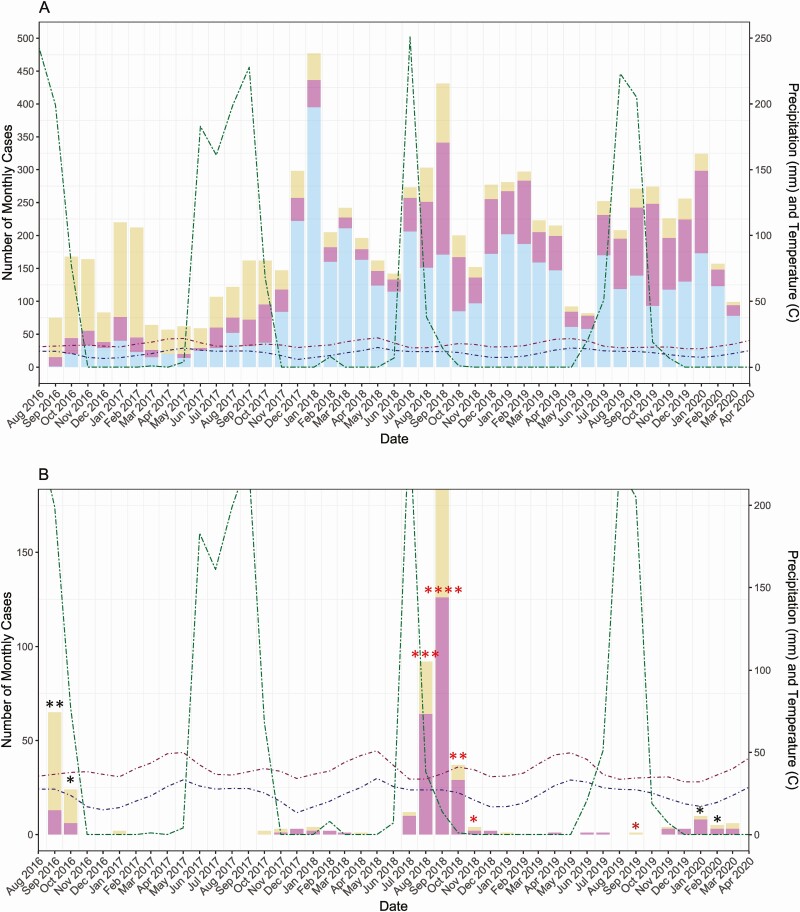
Number of monthly cases of lower respiratory tract infection (LRTI): September 2016-March 2020. Monthly total precipitation in millimeters (mm) and monthly mean high and low temperatures in degrees Celsius (°C) are shown to reflect seasonal patterns of infection. Top to bottom: (A) Number of monthly cases of nonsevere, severe, and very severe LRTI; (B) number of monthly cases of severe and very severe LRTI associated with respiratory syncytial virus (RSV) infection; each asterisk indicates 1 death in the given month. RSV A deaths are indicated in black; RSV B deaths are indicated in red. One death associated with both RSV A and RSV B infection occurred in September 2019, indicated in dark red.

The initial recruitment enrolled 3676 children younger than 2 of years age; over the next 3.5 years, an additional 8458 newborns were enrolled in the study (Supplementary Figure 1). Among these subjects, there were 4683 cases of nonsevere LRTIs, 2064 severe LRTIs, and 1732 very severe LRTIs (Figure 1). Periods where there were relatively fewer cases of nonsevere LRTI appeared to correlate with those where severe or very severe LRTI predominated (Figure 1A), coinciding with the RSV season (Figure 1B). There was an initial RSV A season in 2016 followed by a much larger RSV B season in 2018, and an almost absent RSV A in the following season.

Demographics for the entire cohort are presented in Table 1 and Supplementary Table 2. Of the 12,134 subjects, 6206 (51%) were boys, and 505 died of any cause. There were 16 RSV-associated deaths. Boys were significantly more likely to die of any cause than were girls (*P* = .004). Other risk factors significantly associated with dying were younger maternal and younger paternal ages (*P* < .01) and a higher rate of maternal unemployment (*P* < .001). This trend was also seen in those who died of RSV compared with those who survived; these were not significant likely because of the small number of children who died of RSV resulting in lower power to detect differences.

**Table 1.** Demographics of Study Population by Outcome

Characteristic	Cohort (N = 12 134)	Survived (n = 11 629)	All-cause Deaths (n = 505)	RSV+ Deaths (n = 16)
Male	6206 (51.1%)	5916 (50.9%)^a^	290 (57.4%)^a^	11 (68.8%)
Mean birth weight, g	2659 (SD = 445.9)	2677 (SD = 424.1)^b^	2230 (SD = 678.9)^b^	2671 (SD = 488.0)
Birth place				
Home birth	4002 (33.0%)	3823 (32.9%)	179 (35.4%)	7 (43.8%)
Medically attended birth	8110 (66.8%)	7787 (67.0%)	323 (64.0%)	9 (56.3%)
Child caretaker				
Parents	11 700 (96.4%)	11 229 (96.6%)^b^	471 (93.3%)^b^	16 (100.0%)
Grandparents	368 (3.0%)	364 (3.1%)	4 (0.8%)	0 (0.0%)
Other/not known	66 (0.5%)	36 (0.3%)	30 (5.9%)	0 (0.0%)
Parents				
Mother’s age, years, mean	23.8 (SD = 3.4)	23.8 (SD = 3.4)^a^	23.4 (SD = 3.7)^a^	23.6 (SD = 3.1)
Mother’s education				
Completed primary	5283 (43.5%)	5058 (43.5%)	225 (44.6%)	10 (62.5%)
More than primary level	6739 (55.5%)	6467 (55.6%)	272 (53.9%)	6 (37.5%)
Not known	112 (0.9%)	104 (0.9%)	8 (1.6%)	0 (0.0%)
Mother’s employment				
Employed	4927 (40.6%)	4771 (41.0%)^b^	156 (30.9%)^b^	4 (25.0%)
Not employed	6964 (57.4%)	6624 (57.0%)	340 (67.3%)	12 (75.0%)
Other/unknown	243 (2.0%)	231 (2.0%)	9 (1.8%)	0 (0.0%)
Mother’s employment type				
Unskilled labor	3277 (27.0%)	3178 (27.3%)	99 (19.6%)	5 (31.3%)
Other	1846 (15.2%)	1777 (15.3%)	69 (13.7%)	1 (6.3%)
Not known	7011 (57.8%)	6674 (57.4%)	337 (66.7%)	10 (62.5%)
Father’s age, years, mean	26.8 (SD = 4.0)	26.8 (SD = 3.9)^a^	26.4 (SD = 4.1)^a^	28.4 (SD = 6.3)
Father’s education				
Completed primary	3850 (33.1%)	3688 (31.7%)	162 (32.1%)	7 (43.8%)
More than primary level	8217 (70.7%)	7882 (67.8%)	335 (66.3%)	9 (56.3%)
Not known	67 (0.6%)	59 (0.5%)	8 (1.6%)	0 (0.0%)
Father’s employment				
Employed	11 543 (99.3%)	11 065 (95.2%)	478 (94.7%)	16 (100.0%)
Not employed	452 (3.9%)	432 (3.7%)	20 (4.0%)	0 (0.0%)
Other/unknown	139 (1.2%)	132 (1.1%)	7 (1.4%)	0 (0.0%)
Father’s employment type				
Unskilled labor	6866 (56.6%)	6578 (56.6%)	288 (57.0%)	13 (81.3%)
Other	4775 (39.4%)	4582 (35.9%)	193 (38.2%)	2 (12.5%)
Not known	493 (4.1%)	469 (4.0%)	24 (4.8%)	0 (0.0%)
Household details				
Other children <5 years				
No other children	5659 (46.6%)	5322 (45.8%)	337 (66.7%)	7 (43.8%)
1 other child	4952 (40.8%)	4835 (41.6%)	117 (23.2%)	7 (43.8%)
2 others	1390 (11.5%)	1349 (11.6%)	41 (8.1%)	2 (12.5%)
>2 other	127 (1.0%)	120 (1.0%)	7 (1.4%)	0 (0.0%)
Other children 5–14 years				0 (0.0%)
No other children	10 677 (88.0%)	10 222 (87.9%)	455 (90.1%)	13 (81.3%)
1 other child	941 (7.8%)	915 (7.9%)	26 (5.1%)	2 (12.5%)
2 others	364 (3.0%)	356 (3.1%)	8 (1.6%)	0 (0.0%)
>2 other	146 (1.2%)	133 (1.1%)	13 (2.6%)	1 (6.3%)
Wealth score	-1.6 (SD = 1.6)	-1.6 (SD = 1.6)	-1.7 (SD = 1.5)	-2.1 (SD = 4.2)

^a^*P* < .01.

^b^*P* < .001.

RSV was identified on the NP swabs for 485 infants and children, of whom 472 had an LRTI, 271 had severe LRTIs, 195 had very severe LRTIs, and 6 were collected from children with nonsevere LRTI (misclassified). Twelve children were classified with an upper respiratory tract infection and 1 child with meningitis had a swab collected at the time of death. Five of 271 children age 0–2 years with severe RSV-associated LRTIs, died, as did 10/195 with very severe LRTIs leading to CFRs of 1.8% and 5.1%, respectively (Table 2). All but 1 of these deaths occurred in the community, leading to higher CFRs in the community in all ages. The highest CFRs for severe LRTIs was in the first 3 months of life, which was 9.1% of community severe RSV LRTI. All very severe RSV LRTI deaths occurred in the community with the highest CFR of 28.6%, also occurring in the first 3 months of life.

**Table 2.** Case Fatality Ratio for RSV-associated LRTIs

	Age at Death	RSV Severe LRTI Cases	RSV Severe LRTI Deaths	RSV Severe LRTI Case Fatality Ratio	RSV Very Severe LRTI Cases	RSV Very Severe LRTI Deaths	RSV Very Severe LRTI Case Fatality Ratio
	Days	No.	No.	%	No.	No.	%
Community	0–90	22	2	9.1	14	4	28.6
	0–180	52	3	7.1	34	6	17.6
	0–365	125	3	2.4	68	8	11.8
	0–730	169	4	2.4	101	10	9.9
Hospital associated	0–90	18	0	0.0	23	0	0.0
	0–180	36	1	2.8	46	0	0.0
	0–365	73	1	1.4	71	0	0.0
	0–730	102	1	1.0	94	0	0.0
Overall	0–90	40	2	5.0	37	4	10.8
	0–180	88	4	4.5	76	6	7.9
	0–365	198	4	2.0	134	8	6.0
	0–730	271	5	1.8	195	10	5.1

Abbreviations: LRTI, lower respiratory tract infection; RSV, respiratory syncytial virus.

**Figure 2.**
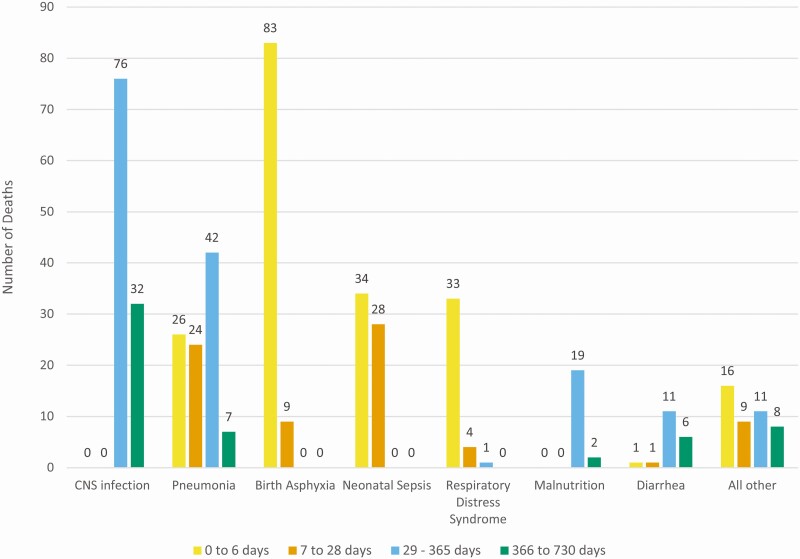
Number of verbal autopsies collected by cause of death. Number of verbal autopsies conducted on all children who died and the most probable cause of death as read by 2 experienced physician readers and adjudicated by a third when disagreement arose in the most probable cause of death. Showing most common classifications: central nervous system (CNS) infection n = 108, pneumonia n = 99, birth asphyxia n = 92, neonatal sepsis n = 62; respiratory distress syndrome n = 38, malnutrition n = 21, diarrhea n = 19, other causes n = 44. The age groups and numbers of adjudicated causes of death are as follows: (A) early neonatal deaths, 0–6 days (N = 192); (B) late neonatal deaths, 7–28 days (N = 75); (C) postneonatal infant deaths, 29–365 days (N = 161); (D) all deaths in children younger than age 2 years, 366–730 days (N = 55).

Of the 489 deaths, a VA was conducted in 483 subjects (Figure 2). Pneumonia, sepsis, or respiratory distress syndrome was the most probable cause of 93/193 (48.1%) early neonatal deaths and for 56/75 (74.7%) of late neonatal deaths. CNS infections (108/483, 22%) and pneumonia (99/483, 20%) were the most important overall causes of death in this younger than age 2 years population. Pneumonia accounted for 26/193 (13.5%) early neonatal, 24/75 (32.0%) late neonatal, 42/160 (26.3%) postneonatal infant deaths, and 7/55 (13%) of 1- to 2-year deaths.

Because this study was a prospective active surveillance of infants and young children for LRTI and mortality, we were able to calculate crude and adjusted population-based estimates of LRTIs and RSV LRTIs (Table 3) and infant and child mortality rates, attributable to LRTIs and RSV LRTIs (Table 4). These rates are also presented during the RSV season (Tables 3, 4). The adjusted mortality rate from RSV LRTIs in the first year of life is 1.6 (0.6–1.7)/1000 CY, the highest rates being in the first 3 months of life: 3.0 (1.4–6.6)/1000 CY. The mortality rates are 7- to 10-fold higher in the community compared with the hospital (Table 3). This accounts for approximately 10% of all LRTI death. The adjusted rate in the RSV season is almost double: 3.1/1000 CY first year of life and 6.1/1000 CY first 3 months of life, and is almost 20% of LRTI deaths (Table 3). The infant mortality in Melghat over the study was 51.3/1000 live births, which increased to 54.4/1000 live births during the RSV season (Table 4), almost 60% occurring in the neonatal period. Overall, RSV did not contribute significantly to the neonatal mortality; however, RSV accounted for 8% of the annual postneonatal infant mortality that rose to 10% in the RSV season.

**Table 3.** Mortality Rates for LRTI and RSV-associated LRTI

			Deaths										
Age at death	CY	Total Deaths	Total LRTI Deaths	LRTI Deaths With Swab Collected	Deaths With RSV+ Swab	RSV+ LRTI Deaths	Crude LRTI Mortality Rate	Adjusted LRTI Mortality Rate	Crude RSV+ Mortality Rate	Adjusted RSV+ Mortality Rate	Proportion of LRTI Deaths That are RSV+	Crude RSV + LRTI Mortality Rate	Adjusted RSV + LRTI Mortality Rate
Days		No.	No.	No.	No.	No.	1000 CY (95% CI)	1000 CY (95% CI)	1000 CY (95% CI)	1000 CY (95% CI)	% (95% CI)	1000 CY (95% CI)	1000 CY (95% CI)
Overall													
0–90	2063.8	344	102	98	6	6	47.5 (57.6–39.2)	49.4 (59.7–40.9)	2.9 (6.5–1.3)	3.0 (6.6–1.4)	6.1 (12.9–2.3)	2.9 (6.5–1.3)	3.0 (6.6–1.4)
0–180	4111.9	381	117	113	10	10	27.5 (33.0–22.9)	28.5 (34.0–23.8)	2.4 (4.5–1.3)	2.5 (4.6–1.4)	8.8 (15.7–4.3)	2.4 (4.5–1.3)	2.5 (4.6–1.4)
0–365	8020.9	433	140	132	13	12	16.5 (19.5–13.9)	17.5 (20.6–14.8)	1.6 (2.8–0.9)	1.7 (2.9–1.0)	9.1 (16.3–5.4)	1.5 (2.6–0.9)	1.6 (2.7–0.9)
0–730	15 525.7	489	152	144	16	15	9.3 (10.9–7.9)	9.8 (11.5–8.4)	1.0 (1.7–0.6)	1.1 (1.8–0.7)	10.4 (17.4–6.5)	1.0 (1.6–0.6)	1.0 (1.7–0.6)
Community													
0–90	2063.8	269	90	87	6	6	42.2 (51.8–34.3)	43.6 (53.4–35.6)	2.9 (0.6–0.1)	3.0 (0.7–0.1)	6.9 (14.4–2.6)	2.9 (6.5–1.3)	3.0 (6.6–1.4)
0–180	4111.9	302	100	95	9	9	23.1 (28.2–18.9)	24.3 (29.5–20.0)	2.2 (0.4–0.1)	2.3 (0.4–0.1)	9.5 (17.2–4.4)	2.2 (4.2–1.1)	2.3 (4.4–1.2)
0–365	8020.9	344	119	111	12	11	13.8 (16.6–11.5)	14.8 (17.7–12.4)	1.5 (0.3–0.1)	1.6 (0.3–0.1)	9.9 (17.0–6.4)	1.4 (2.6–0.9)	1.5 (0.0–0.0)
0–730	15 525.7	388	127	122	15	14	7.9 (9.4–6.6)	8.2 (9.7–6.9)	1.0 (0.2–0.1)	1.0 (0.2–0.1)	11.5 (18.5–0.0)	0.9 (1.5–0.5)	0.9 (0.0–0.0)
Hospital													
0–90	2063.8	75	12	11	0	0	5.3 (9.6–3.0)	5.8 (10.2–3.3)	0.0 (0.0–0.0)	0.0 (0.0–0.0)	0.0 (0.0–0.0)	0.0 (0.0–0.0)	0.0 (0.0–0.0)
0–180	4111.9	82	17	16	1	1	3.9 (6.3–2.4)	4.1 (6.6–2.6)	0.2 (0.2–0.0)	0.3 (0.2–0.0)	6.3 (30.2–0.2)	0.2 (1.7–0.0)	0.3 (1.7–0.0)
0–365	8020.9	89	21	18	1	1	2.2 (3.6–1.4)	2.6 (4.0–1.7)	0.1 (0.1–0.0)	0.1 (0.1–0.0)	5.6 (27.3–0.1)	0.1 (0.9–0.0)	0.1 (0.9–0.0)
0–730	15 525.7	101	25	22	1	1	1.4 (2.2–0.9)	1.6 (2.4–1.1)	0.1 (0.0–0.0)	0.1 (0.0–0.0)	4.5 (22.8–0.1)	0.1 (0.5–0.0)	0.1 (0.5–0.0)
RSV season													
0–90	1040.8	225	63	60	6	6	57.6 (73.7–45.1)	60.5 (76.9–47.6)	5.8 (12.8–2.6)	6.1 (13.2–2.8)	10.0 (20.5–3.8)	5.8 (12.8–2.6)	6.1 (13.2–2.8)
0–180	2152.7	225	73	70	10	10	32.5 (40.9–25.8)	33.9 (42.5–27.1)	4.6 (8.6–2.5)	4.8 (8.9–2.6)	14.3 (24.7–7.1)	4.6 (8.6–2.5)	4.8 (8.9–2.6)
0–365	4197.7	287	88	81	13	12	19.3 (23.9–15.6)	21.0 (25.8–17.0)	3.1 (5.3–1.8)	3.4 (5.7–2.0)	14.8 (0.0–0.0)	2.9 (0.0–0.0)	3.1 (5.3–1.8)
0–730	8270.0	326	97	90	16	15	10.9 (13.4–8.9)	11.7 (14.3–9.6)	1.9 (3.2–1.2)	2.1 (3.3–1.3)	16.7 (0.0–0.0)	1.8 (3.0–1.1)	2.0 (3.2–1.2)
Community													
0–90	1040.8	179	55	53	7	6	50.9 (66.2–39.2)	52.8 (6.8–4.1)	5.8 (1.3–0.3)	6.0 (1.3–0.3)	11.3 (23.0–4.3)	5.8 (12.8–2.6)	6.0 (1.3–0.3)
0–180	2152.7	205	63	61	9	9	28.3 (36.3–22.1)	29.3 (3.7–2.3)	4.2 (0.9–0.3)	4.3 (0.9–0.3)	14.8 (26.2–7.0)	4.2 (8.0–2.2)	4.3 (0.8–0.2)
0–365	4197.7	232	75	71	12	11	16.9 (21.3–13.4)	17.9 (2.2–1.4)	2.9 (0.5–0.2)	3.0 (0.6–0.2)	15.5 (26.0–8.0)	2.6 (4.7–1.5)	2.8 (0.5–0.2)
0–730	8270.0	262	81	77	15	14	9.3 (11.6–7.5)	9.8 (1.2–0.8)	1.8 (0.3–0.1)	1.9 (0.3–0.1)	18.2 (28.6–10.3)	1.7 (2.9–1.0)	1.8 (0.3–0.1)
Hospital													
0–90	1040.8	46	8	7	0	0	6.7 (14.1–3.2)	7.7 (1.5–0.4)	0.0 (0.0–0.0)	0.0 (0.0–0.0)	0.0 (0.0–0.0)	0.0 (0.0–0.0)	0.0 (0.0–0.0)
0–180	2152.7	50	10	9	1	1	4.2 (8.0–2.2)	4.6 (0.9–0.3)	0.5 (0.3–0.0)	0.5 (0.3–0.0)	11.1 (48.3–0.3)	0.5 (3.3–0.1)	0.5 (0.3–0.0)
0–365	4197.7	55	13	10	1	1	2.4 (4.4–1.3)	3.1 (0.5–0.2)	0.2 (0.2–0.0)	0.3 (0.2–0.0)	10.0 (44.5–0.3)	0.2 (1.7–0.0)	0.3 (0.2–0.0)
0–730	8270.0	64	16	13	1	1	1.6 (2.7–0.9)	1.9 (0.3–0.1)	0.1 (0.1–0.0)	0.1 (0.1–0.0)	7.7 (36.0–0.2)	0.1 (0.9–0.0)	0.1 (0.1–0.0)

Abbreviations: CY, child years of observation; RSV LRTI, respiratory syncytial virus lower respiratory infection; RSV+, nasal swab tested positive for respiratory syncytial virus by polymerase chain reaction.

^a^Deaths with history of LRTI or verbal autopsy cause of death of pneumonia and RSV+ swab.

**Table 4.** Neonatal and Infant Mortality Rates and Attributable to Lower Respiratory Tract Infections and RSV

	All-cause Mortality Rates		LRTI Mortality Rates		Crude RSV + LRTI Mortality Rates		Adjusted RSV + LRTI Mortality Rates	
	Overall	During RSV Season	Overall	During RSV Season	Overall	During RSV Season	Overall	During RSV Season
	/1000 LB/Year	/1000 LB/Year	/1000 LB/Year	/1000 LB/Year	/1000 LB/Year	/1000 LB/Year	/1000 LB/Year	/1000 LB/Year
Early neonatal	23.0	22.6	6.3	5.7	0.0	0.0	0.0	0.0
Late neonatal	8.9	8.5	2.4	2.3	0.1	0.2	0.1	0.2
Neonatal	31.8	31.1	8.6	8.0	0.1	0.2	0.1	0.2
Infant	51.3	54.4	7.9	18.4	1.5	2.3	1.6	2.5

Abbreviations: LB, live births; LRTI, lower respiratory tract infections; RSV, respiratory syncytial virus.

## Discussion

This study, which enrolled 12 134 newborns, infants, and children and followed them with active surveillance for the first 2 years of life, conducted in a poor rural community with poor access to care, showed that 93% of RSV LRTI-related deaths occurred in the community rather than in a hospital. The case fatality rate in the community for very severe RSV LRTI was between 5 and 7 times higher than severe RSV LRTI, justifying its continued utility in classifying severe LRTI disease. In the first 3 months of life, almost 30% of babies with very severe RSV LRTIs died compared with 5% with severe disease. In contrast, none of the children admitted to the hospital with very severe RSV LRTIs died, whereas the CFR from severe RSV LRTIs in the hospital was similar to those in the community. That infants and children with very severe RSV LRTIs survived in the hospital, but had a very high CFR in the community, leads to the conclusion that hospital care with intravenous fluids antibiotics and oxygen (the only available treatment modalities available at district hospitals in Melghat) helped these babies survive. Only 1 infant with severe RSV LRTI died in the hospital, 11 days after admission.

Because this was an active surveillance for LRTI and all-cause mortality in the community and in the healthcare facilities serving this community of 93 villages, the study allowed for accurate assessments of rates per thousand child years of observation. The adjusted RSV LRTI mortality rates are marginally higher than the crude rates, attesting to the fact that the VHWs and counsellors obtained nasal swabs from the majority of infants and children with LRTIs who died in the community or the health facility. The adjusted RSV LRTI mortality/1000 CY was highest in the first 3 months of life, marginally different than in the first 6 months (3.0 and 2.5, respectively). Most of this was attributed to mortality in the community.

Current global burden of disease estimates for RSV rely mostly on adjustment of hospital-based CFR to the community with an inflation factor of 1.5, 2.1, or 2.9 [1] based on 3 studies from Argentina [13], Bangladesh [14], and Indonesia [15, 16], the only data available at the time. In that study, the meta-estimate of the CFR was 1.9 for children 0–5 years of age [1], similar to our estimate of 1.8 for children <2 years. This estimate (1.9 for younger than age 5 years) belies the much higher CFR for younger age groups, especially for RSV where the highest mortality is in the first 6 months of life. Our study would suggest that, in areas with a high infant mortality where access to care is poor, the inflation factor may be much higher for community deaths.

With the distinct possibility of maternal immunization with RSV vaccines [17] or long-acting monoclonal antibodies [18], to prevent morbidity in infants between 3 and 6 months of age, becoming a viable strategy for preventing RSV LRTI deaths in low middle income countries [19], these numbers become very significant. Two strategies could be applied: seasonal vaccination as with influenza virus or year-round vaccination [20]. To inform policy, we then calculated adjusted RSV LRTI mortality rates during the RSV season. Not surprisingly, the point estimates of these rates almost doubled. Recognizing that seasonal data for RSV are not available in many of the most populous lower middle-income countries (LMIC) contributing to the global burden of disease, the WHO has initiated RSV surveillance in more than 20 LMICs, piggybacked on routine influenza surveillance [21]. Given the year-to-year variability in RSV seasons in some countries [22, 23], and the difficulty of implementing seasonal vaccination of pregnant women and the campaigns that will be necessary in LMIC, data such as these can be used to assist in policymaking and economic disease modelling [24].

Other common metrics applied for decision-making in LMIC are the neonatal, postneonatal infant mortality, and infant mortality rates per thousand live births [25]. These rates are useful as they are an indirect indicator, of both the socioeconomic health of the population and the access to quality healthcare. In Melghat, the infant mortality of >50 is comparable to that of several low-income countries, and when examined at the district level, is equivalent to a significant proportion of the poorer districts in India [26, 27]. Although the community and hospital case fatality rates are useful for extrapolating the rates per thousand child years for epidemiologic and economic comparisons, active surveillance for RSV LRTIs in the community and hospital allows for a direct estimate of the RSV LRTIs attributable fraction of the neonatal, and infant mortality. Not surprisingly, RSV does not contribute significantly to neonatal mortality in this community-based study, probably because of transplacentally transferred maternal antibodies; [28, 29] however, it is a significant contributor to postneonatal infant mortality accounting for 7.3% overall and 10% in the RSV season. RSV LRTI also accounts for 20% of the overall LRTI mortality in infancy, equivalent to the rates in the Bangladesh and Indonesian community studies [14, 15]. These data are derived from a typical rural area, in many LMIC without easy access to health facilities with ventilatory assistance for young babies and district hospitals, and would arguably be applicable to most such situations where access to care is poor.

In the study area, VHWs were available all the time (except when they were out of the village). Free transportation to the nearest hospital and all of the charity hospitals was offered to every family in the village. Free care was available at the hospitals (both government as well as the charity hospital), and there were other incentives, including educational, social, economic, and other developmental activities in place by the MAHAN trust; despite this, 14 of 15 LRTI deaths occurred at home. Clearly, a significant number of these deaths could have been prevented (there were no very severe RSV LRTI deaths in the hospital); despite all of the barriers to access being potentially breached, families did not access care. In fact, 6 children died in the hospital, 4 of them on the day of admission, and by our definition were considered to be community deaths because they were taken to the hospital in extremis.

One of the main limitations of this study, it might be argued, is that the attribution of the LRTI death was based on obtaining an NP specimen from a child with LRTI who died, not directly from the lung, as done in the CHAMPS project. The CHAMPS project has focused on hospital-based deaths, which are mostly in children with complex disease and include tertiary care hospitals catering to children with good access to care in an urban setting [3]. Recognizing this weakness, our team has embarked on obtaining lung tissue postmortem using minimally invasive tissue sampling [30], in the same villages and hospitals without ventilator support. Of necessity, neither minimally invasive tissue sampling nor CHAMPS will be performed on all children who die in the community. On the other hand, we were able to obtain NP swabs on 95% of children with LRTIs who died. In the end, it will probably only be probe studies with maternal immunization or monoclonal antibodies that would be able to truly address the preventable fraction of mortality resulting from RSV.

In conclusion, this population-based active surveillance study found 6.5 times higher rates of community mortality from RSV than hospital rates, emphasizing the need for community-based studies in burden of disease estimates.

## Supplementary Data

Supplementary materials are available at *Clinical Infectious Diseases* online. Consisting of data provided by the authors to benefit the reader, the posted materials are not copyedited and are the sole responsibility of the authors, so questions or comments should be addressed to the corresponding author.

ciab481_suppl_Supplementary_Material

## Notes

***Acknowledgments.*** We thank Niteen Wairagkar, Padmini Srikantiah, and Prachi Vora from the Bill and Melinda Gates Foundation for their input and guidance at every stage of this study; members of the steering committee Shabir A. Madhi, Dean, Faculty of Health Sciences, University of the Witwatersrand, Johannesburg, South Africa; Sanjay Mehendale, Director Research, PD Hinduja Hospital and Medical Centre, Pune, India, former Additional Director General, Indian Council of Medical Research New Delhi India; Sanjay Zodpey, Vice President—Academics, Public Health Foundation of India, New Delhi, Director—Indian Institute of Public Health, Delhi; and Subhash R. Salunke, Senior Advisor, PHFI and Officiating Director, IIPH-Bhubaneswar and former Director General Health Services of Maharashtra State. We would like to thank the MAHAN trust foundation, Kavita Satav, staff and data entry team, all of the study team, the supervisors, counsellors, and village health workers who worked tirelessly through the study. Dr Shinde was the pediatrician that took care of our study subjects at the MAHAN Hospital and was one of the readers of the verbal autopsies. The sincere efforts of the National Influenza Centre technical staff at the National Institute of Virology are acknowledged. We acknowledge with gratutude the assistance of the Hon. District Collector and Additional District Medical Officer Dr Bobade, the pediatricians at the subdistrict hospital, the 93 village Panchayat Heads and Council Members, Dr Shubhada Khirwadakr, Dr Abhijit Bharadwaj, Dr Deoghare, and Dr Patil who taught all our supervisors and counsellors as well as the local traditional healers and auxillary nurse midwives in grief counselling and provided grief counselling; and all the traditional midwives and auxillary midwives who assisted with sample collection and grief counselling. We acknowledge Dr Elisabeth Root, Ohio State University, who set up the Geographic Information System at the site and Robert Suss, University of Colorado, for assistance with figures. Finally and most importantly we acknowledge with gratitude all of the families and their young children that participated in the study who graciously let us into their homes and allowed us to follow their babies throughout the study and especially those families with babies that died, to whom we could offer no solace, except to know that their participation will help other babies in the future from dying of preventable causes.

***Supplement sponsorship.*** This supplement is sponsored by the Bill and Melinda Gates Foundation.

***Financial support.*** Bill and Melinda Gates foundation OPP1128468, Caring Friends and Bajaj Holdings and Investment Department.

***Potential conflicts of interest.*** E.S. reports grants to institution, personal fees, and travel fees from AstraZeneca Inc, Merck & Co, Regeneron Inc, Pfizer Inc, and Roche Inc; consultation, lecture fees, travel support, and data and safety monitoring board fees from Abbvie Inc; data and safety monitoring board fees from GSK Inc; consultation fees from Alere Inc; grants to institution from Johnson and Johnson; and grants to institution and travel support from Novavax Inc, outside the submitted work. All other authors have no potential conflicts. All authors have submitted the ICMJE Form for Disclosure of Potential Conflicts of Interest. Conflicts that the editors consider relevant to the content of the manuscript have been disclosed.
