# Supplementary material for: Mortality From Respiratory Syncytial Virus in Children Under 2 Years of Age: A Prospective Community Cohort Study in Rural Maharashtra, India
Source: Clin Infect Dis. 2021 Sep 2;73(Suppl 3):S193–202. doi: 10.1093/cid/ciab481 (PMC8411248; doi:10.1093/cid/ciab481)

| **Supplemental Table 1: Study Definitions** | |
| --- | --- |
| Term | Definition |
| *Respiratory Illness episode:* | Since a sick child could have been seen by a village health worker or a supervisor at their home or by a counselor or a physician at the health center or hospital on admission or have been seen by a village health worker 7-day follow-up visit, we defined a composite illness episode encompassing the 15 days starting with the first contact with any of the study staff in the context of an ill visit. At each of these visits a child could potentially have had a record of danger signs, chest wall and drawing or tachypnea; the severity of the illness was the most severe classification of the illness at any of these contacts. |
| *Very Severe LRTI:* | A child with World Health Organization (WHO) defined general danger signs: inability to feed, lethargy unconsciousness, convulsions or vomiting everything. |
| *Severe LRTI:* | A child with cough or difficult breathing without general danger signs but with the presence of lower chest wall in drawing. |
| *Nonsevere LRTI*: | A child with cough or difficult breathing with WHO defined tachypnoea (an observed respiratory rate of: > 60 beats per minute for an infant <59 days of age, > 50 beats per minute for an infant 60-365 days of age, and > 40 per minute for a child 365-720 days of age. |
| *No LRTI/URTI*: | A child with cough or difficult breathing without general danger signs, chest wall and drawing or tachypnea. |
| *LRTI Death:* | A child with and LRTI in the 15 days preceding death meeting one of the above criteria if the child was seen by one of the study staff or was diagnosed to have an LRI or pneumonia by a physician at the hospital. In the absence of an illness visit preceding the death, a verbal autopsy diagnosis of pneumonia classified the death as an LRTI death. |

| **Supplementary Table 2. Durable Assets and Housing Characteristics of Study Population** | | | | | | | | | | |
| --- | --- | --- | --- | --- | --- | --- | --- | --- | --- | --- |
|  | **Cohort n=12134** | | **Survived n=11629** | | **All Cause Deaths n=505** | | | | **RSV+ Deaths n=16** | |
| **WEALTH SCORE** | -1.6 | (SD=1.6) | -1.6 | (SD=1.6) | -1.7 | | (SD=1.5) | -2.1 | | (SD=4.2) |
| **Characteristics** | | | | | | | | | | |
| **House ownership** |  |  |  |  |  |  | | |  |  |
| Own | 12,052 | (99.3%) | 11,552 | (99.3%) | 500 | (99.0%) | | | 16 | (100.0%) |
| Rent | 52 | (0.4%) | 52 | (0.4%) | 0 | (0.0%) | | | 0 | (0.0%) |
| Other | 24 | (0.2%) | 22 | (0.2%) | 2 | (0.4%) | | | 0 | (0.0%) |
| Not known | 6 | (0.0%) | 3 | (0.0%) | 3 | (0.6%) | | | 0 | (0.0%) |
| **Electricity** |  |  |  |  |  |  | | |  |  |
| yes, metered/own | 4,195 | (34.6%) | 4,022 | (34.6%) | 173 | (34.3%) | | | 3 | (18.8%) |
| yes, but no meter | 4,532 | (37.3%) | 4,342 | (37.3%) | 190 | (37.6%) | | | 9 | (56.3%) |
| no electricity | 3,401 | (28.0%) | 3,262 | (28.1%) | 139 | (27.5%) | | | 4 | (25.0%) |
| **Owns land** | 5,886 | (48.5%) | 5,631 | (48.4%) | 248 | (49.1%) | | | 7 | (43.8%) |
| **Floor material** |  |  |  |  |  |  | | |  |  |
| Mud/cow dung | 11,367 | (93.7%) | 10,899 | (93.7%) | 468 | (92.7%) | | | 16 | (100.0%) |
| Tile | 509 | (4.2%) | 485 | (4.2%) | 24 | (4.8%) | | | 0 | (0.0%) |
| Wood | 228 | (1.9%) | 220 | (1.9%) | 8 | (1.6%) | | | 0 | (0.0%) |
| Other | 30 | (0.2%) | 25 | (0.2%) | 5 | (1.0%) | | | 0 | (0.0%) |
| **Roof material** |  |  |  |  |  |  | | |  |  |
| Tin | 7,990 | (65.8%) | 7,661 | (65.9%) | 329 | (65.1%) | | | 8 | (50.0%) |
| Kavelu | 2,648 | (21.8%) | 2,537 | (21.8%) | 111 | (22.0%) | | | 4 | (25.0%) |
| Thatch | 1,133 | (9.3%) | 1,084 | (9.3%) | 49 | (9.7%) | | | 4 | (25.0%) |
| Cement | 180 | (1.5%) | 172 | (1.5%) | 8 | (1.6%) | | | 1 | (6.3%) |
| Wood | 161 | (1.3%) | 156 | (1.3%) | 5 | (1.0%) | | | 0 | (0.0%) |
| Asbestos | 15 | (0.1%) | 15 | (0.1%) | 0 | (0.0%) | | | 0 | (0.0%) |
| Other | 7 | (0.1%) | 4 | (0.0%) | 3 | (0.6%) | | | 0 | (0.0%) |
| **Paint type** |  |  |  |  |  |  | | |  |  |
| Chalk | 7,263 | (59.9%) | 6,975 | (60.0%) | 288 | (57.0%) | | | 11 | (68.8%) |
| None | 4,260 | (35.1%) | 4,071 | (35.0%) | 189 | (37.4%) | | | 5 | (31.3%) |
| Synthetic | 494 | (4.1%) | 472 | (4.1%) | 22 | (4.4%) | | | 0 | (0.0%) |
| Other | 105 | (0.9%) | 102 | (0.9%) | 3 | (0.6%) | | | 0 | (0.0%) |
| Unknown | 6 | (0.0%) | 6 | (0.1%) | 0 | (0.0%) | | | 0 | (0.0%) |
| **Source of drinking water** |  |  |  |  |  |  | | |  |  |
| Tap water (public) | 4,875 | (24.6%) | 4,635 | (39.9%) | 240 | (47.5%) | | | 8 | (50.0%) |
| Tap water (own) | 2,986 | (24.6%) | 2,875 | (24.7%) | 111 | (22.0%) | | | 3 | (18.8%) |
| Hand pump/bore well (public) | 2,254 | (18.6%) | 2,167 | (18.6%) | 87 | (17.2%) | | | 5 | (31.3%) |
| Well (public) | 1,716 | (0.7%) | 1,664 | (14.3%) | 52 | (10.3%) | | | 0 | (0.0%) |
| Hand pump/bore well (own) | 101 | (0.8%) | 94 | (0.8%) | 7 | (1.4%) | | | 0 | (0.0%) |
| Well (own) | 85 | (40.2%) | 83 | (0.7%) | 2 | (0.4%) | | | 0 | (0.0%) |
| River | 62 | (0.5%) | 60 | (0.5%) | 2 | (0.4%) | | | 0 | (0.0%) |
| Water piped | 44 | (14.1%) | 43 | (0.4%) | 1 | (0.2%) | | | 0 | (0.0%) |
| lake | 5 | (0.0%) | 5 | (0.0%) | 0 | (0.0%) | | | 0 | (0.0%) |
| **Water purification method** | |  |  |  |  |  | | |  |  |
| Chlorination | 7,465 | (61.5%) | 7,152 | (61.5%) | 313 | (62.0%) | | | 10 | (62.5%) |
| None | 2,055 | (16.9%) | 1,965 | (16.9%) | 90 | (17.8%) | | | 2 | (12.5%) |
| Boiling | 1,309 | (10.8%) | 1,247 | (10.7%) | 62 | (12.3%) | | | 3 | (18.8%) |
| Water filter | 1,195 | (9.8%) | 1,160 | (10.0%) | 35 | (6.9%) | | | 1 | (6.3%) |
| Bottled water | 11 | (0.1%) | 10 | (0.1%) | 1 | (0.2%) | | | 0 | (0.0%) |
| Other | 93 | (0.8%) | 92 | (0.8%) | 1 | (0.2%) | | | 0 | (0.0%) |
| **Home has a toilet facility** | 1,117 | (9.2%) | 997 | (8.6%) | 108 | (21.4%) | | | 12 | (75.0%) |
| **Fuel used for cooking** |  |  |  |  |  |  | | |  |  |
| Wood | 11,536 | (95.1%) | 11,052 | (95.0%) | 484 | (95.8%) | | | 16 | (100.0%) |
| Gas | 496 | (4.1%) | 480 | (4.1%) | 16 | (3.2%) | | | 0 | (0.0%) |
| Cow dung | 76 | (0.6%) | 74 | (0.6%) | 2 | (0.4%) | | | 0 | (0.0%) |
| Kerosene | 11 | (0.1%) | 11 | (0.1%) | 0 | (0.0%) | | | 0 | (0.0%) |
| Electricity | 6 | (0.0%) | 6 | (0.1%) | 0 | (0.0%) | | | 0 | (0.0%) |
| Coal | 2 | (0.0%) | 2 | (0.0%) | 0 | (0.0%) | | | 0 | (0.0%) |
| Other/unknown | 1 | (0.0%) | 1 | (0.0%) | 3 | (0.6%) | | | 0 | (0.0%) |
| **Household Disposable Goods** | |  |  |  |  |  | | |  |  |
| Color tv | 4,919 | (40.5%) | 4,744 | (40.8%) | 175 | (34.7%) | | | 6 | (37.5%) |
| Fan | 4,370 | (36.0%) | 4,206 | (36.2%) | 164 | (32.5%) | | | 4 | (25.0%) |
| Motorbike | 2,147 | (17.7%) | 2,080 | (17.9%) | 67 | (13.3%) | | | 3 | (18.8%) |
| Watch | 2,138 | (17.6%) | 2,069 | (17.8%) | 69 | (13.7%) | | | 5 | (31.3%) |
| Bicycle | 1,352 | (11.1%) | 1,307 | (11.2%) | 45 | (8.9%) | | | 1 | (6.3%) |
| Cellphone | 1,202 | (9.9%) | 1,157 | (9.9%) | 45 | (8.9%) | | | 0 | (0.0%) |
| Water pump | 442 | (3.6%) | 426 | (3.7%) | 16 | (3.2%) | | | 0 | (0.0%) |
| Fridge | 365 | (3.0%) | 353 | (3.0%) | 12 | (2.4%) | | | 0 | (0.0%) |
| Radio | 211 | (1.7%) | 203 | (1.7%) | 8 | (1.6%) | | | 0 | (0.0%) |
| Tractor | 167 | (1.4%) | 161 | (1.4%) | 6 | (1.2%) | | | 1 | (6.3%) |
| Computer | 133 | (1.1%) | 129 | (1.1%) | 4 | (0.8%) | | | 0 | (0.0%) |
| Home phone | 130 | (1.1%) | 124 | (1.1%) | 6 | (1.2%) | | | 3 | (18.8%) |
| Washer | 80 | (0.7%) | 76 | (0.7%) | 4 | (0.8%) | | | 0 | (0.0%) |
| Car | 80 | (0.7%) | 72 | (0.6%) | 8 | (1.6%) | | | 1 | (6.3%) |
| **Livestock** |  |  |  |  |  |  | | |  |  |
| Owns chickens | 5,248 | (43.3%) | 5049 | (43.4%) | 199 | (39.4%) | | | 9 | (56.3%) |
| Owns goats | 3,787 | (31.2%) | 3639 | (31.3%) | 148 | (29.3%) | | | 4 | (25.0%) |
| Owns bullock cart | 3,759 | (31.0%) | 3592 | (30.9%) | 167 | (33.1%) | | | 7 | (43.8%) |
| Owns cows/buffalo/ox | 6910 | (56.9%) | 6603 | (56.8%) | 307 | (60.8%) | | | 12 | (75.0%) |

**Supplementaary Figure 1. Study Design**


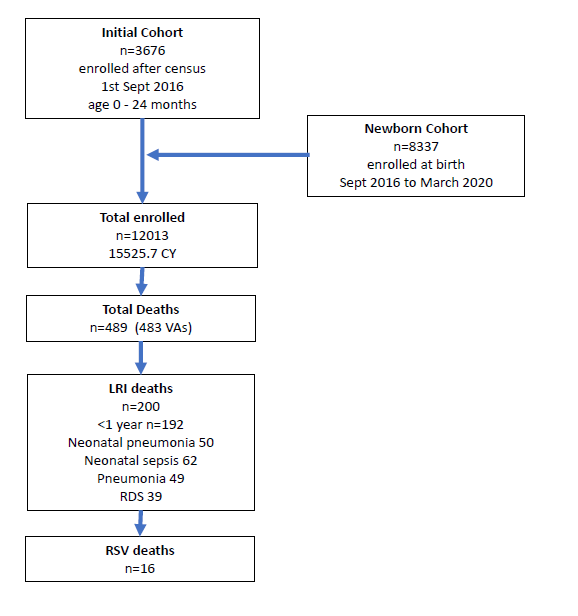

Supplement: ciab481_suppl_Supplementary_Material [file ciab481_suppl_supplementary_material.docx]
